# Synthesis, Characterization, and Computational Modeling of *N*-(1-Ethoxyvinyl)pyridinium Triflates, an Unusual Class of Pyridinium Salts

**DOI:** 10.3390/molecules23020413

**Published:** 2018-02-14

**Authors:** Jonathan D. Shapiro, Justin C. Sonberg, Benjamin C. Schafer, Christopher C. Williams, Hannah R. Ferris, Eric W. Reinheimer, Adam W. Van Wynsberghe, Charles E. Kriley, Max M. Majireck

**Affiliations:** 1 Chemistry Department, Hamilton College, 198 College Hill Road, Clinton, NY 13323, USA; shapiroj18@gmail.com (J.D.S.); jsonberg@hamilton.edu (J.C.S.); justbschafe@gmail.com (B.C.S.); ch.cwilliams03@gmail.com (C.C.W.); hrferris17@gmail.com (H.R.F.); avanwyns@hamilton.edu (A.W.V.W.); 2 Rigaku Oxford Diffraction, 9009 New Trails Drive, The Woodlands, TX 77381, USA; Eric.Reinheimer@rigaku.com; 3 Department of Chemistry, Grove City College, 100 Campus Drive, Grove City, PA 16127, USA; cekriley@gcc.edu

**Keywords:** pyridinium salt, *N*-(1-alkoxyvinyl), *N*,*O*-ketene acetal, ethoxyacetylene, triflic acid, quaternary ammonium compound, hydrogen bonding, X-ray diffractions, crystal structure

## Abstract

*N*-Substituted pyridinium salts constitute one of the most valuable reagent classes in organic synthesis, due to their versatility and ease of use. Herein we report a preliminary synthesis and detailed structural analysis of several *N*-(1-ethoxyvinyl)pyridinium triflates, an unusual class of pyridinium salts with potentially broad use as a reagent in organic synthesis. Treatment of pyridines with trifluoromethane sulfonic acid and ethoxyacetylene generates stable, isolable adducts which have been extensively characterized, due to their novelty. Three-dimensional structural stability is perpetuated by an array of C–H•••O hydrogen bonds involving oxygen atoms from the –SO_3_ groups of the triflate anion, and hydrogen atoms from the aromatic ring and vinyl group of the pyridinium cation. Predictions from density functional theory calculations of the energy landscape for rotation about the exocyclic C–N bond of 2-chloro-1-(1-ethoxyvinyl)pyridine-1-ium trifluoromethanesulfonate (**7**) and 1-(1-ethoxyvinyl)pyridine-1-ium trifluoromethanesulfonate (**16**) are also reported. Notably, the predicted global energy minimum of **7** was nearly identical to that found within the crystal structure.

## 1. Introduction

### Biological and Synthetic Significance of N-Substituted Pyridinium Salts

Quaternary ammonium compounds constitute one of the largest classes of antimicrobial agents known, with *N*-substituted pyridinium salts representing a major subclass [1,2,3,4]. Germicidal properties exhibited by *N*-substituted pyridinium salts cover a broad spectrum of activities, including antiviral, antibacterial, and antifungal. While numerous synthetic approaches exist for producing antimicrobial compounds within this class [4,5,6], widespread drug resistance of clinically relevant microbes, such as the ESKAPE pathogens [7,8,9], ensures that new and efficient synthetic routes to novel structures in this class will be in constant demand.

Beyond their broad spectrum of biological activities, *N*-substituted pyridinium salts hold correspondingly broad potential as reagents in organic synthesis [4,5,6,10,11,12]. For example, Mukaiyama’s reagent (2-chloro-1-methylpyridinium iodide) and numerous onium salt derivatives have been utilized in the synthesis of esters, thioesters, and amides through both inter- and intramolecular dehydrations of carboxylic acids [13,14,15,16,17,18,19,20]. Notably, reagents in this class are generally effective for the synthesis of macrocyclic lactones [21,22,23] and lactams [24,25,26,27,28], but also ring-strained β-lactones [29] and β-lactams [30,31] from acyclic precursors. Furthermore, ketene generation, C–N bond formation, rearrangements, hydroxyl activation, and a variety of other modes of reactivity, have been reported [13,14,15].

## 2. Results and Discussion

### 2.1. Discovery of 2-Chloro-N-(1-ethoxyvinyl)pyridine-1-ium Triflate

**Scheme 1:**
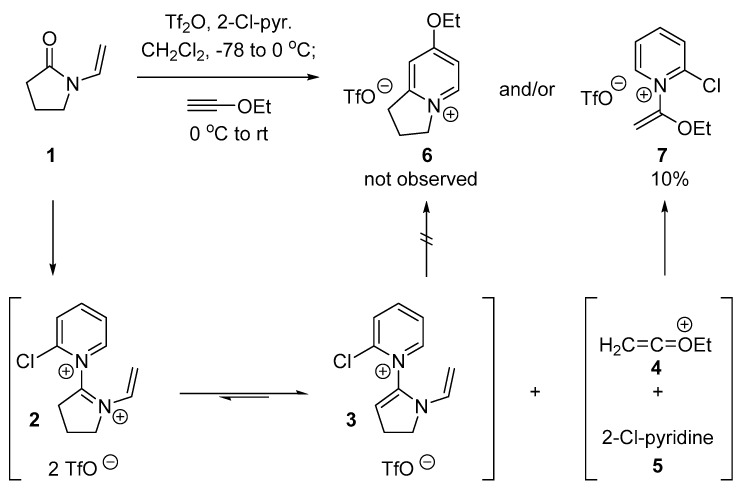
Synthesis of 2-chloro-1-(1-ethoxyvinyl)pyridine-1-ium trifluoromethanesulfonate (**7**) as a side product.

Our interest in the synthesis of antimicrobial *N*-alkylpyridinium alkaloids led us to explore the possibility of utilizing a formal [4 + 2]-cycloaddition approach from readily accessible *N*-vinyl amides, as shown in Scheme 1. In this exploratory reaction, we sought to examine the possibility that the embedded 2-aza-diene within the highly reactive *N*-vinyl iminium **2**, produced via triflic anhydride-promoted amide activation [32,33,34] of *N*-vinylpyrrolidinone (NVP, **1**), would exist long enough to react with ethoxyacetylene to provide pyridinium triflate **6** via a [4 + 2]-cycloaddition/elimination cascade [35,36]. However, in initial experiments, the desired product **6** was not observed. This is likely because the transient dication intermediate **2**, if generated, would rapidly convert to *N*-vinyl enamine **3**, allowing for undesired side reactions to take place [37]. Instead, we observed that adduct **7**, resulting from addition of 2-chloropyridine (**5**) to ketenium intermediate **4**, was cleanly isolated from the crude product mixture in 10% yield.

**Figure 1:**
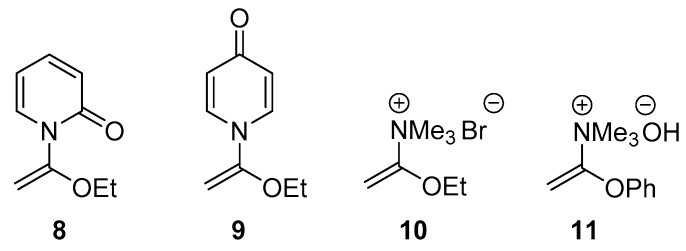
Previously reported *N*-(1-ethoxyvinyl) pyridones and representative *N*-(1-alkoxyvinyl) and *N*-(1-aryloxyvinyl) ammonium salts.

To the best of our knowledge, there are no reports on the synthesis, characterization, and reactivity of *N*-(1-alkoxyvinyl)pyridinium salts. In the most structurally similar examples, Weinstein reported a thermally promoted addition of ethoxyacetylene to 2- and 4-pyridone to yield neutral adducts **8** and **9**, respectively, following a 12–27 d reflux period using excess ethoxyacetylene (Figure 1) [38]. Limited attempts to utilize these potentially valuable compounds in [4 + 2]-cycloadditions were unsuccessful, however, and no follow-up studies on the structure and reactivity of these interesting species are available. While numerous studies have been published on the synthesis and reactivity of neutral ketene *N*,*O*-acetals [39] like **8** and **9**, only scattered reports exist of *N*-quaternized ketene *N*,*O*-acetals (*N*-(1-alkoxyvinyl)ammonium salts) [40,41,42,43,44,45,46,47,48], such as compounds **10** and **11**. Furthermore, general methods of synthesis and systematic evaluation of reactivity within this potentially valuable compound class are lacking.

### 2.2. Optimized Synthesis of N-(1-Ethoxyvinyl)pyridinium Triflates

**Scheme 2:**
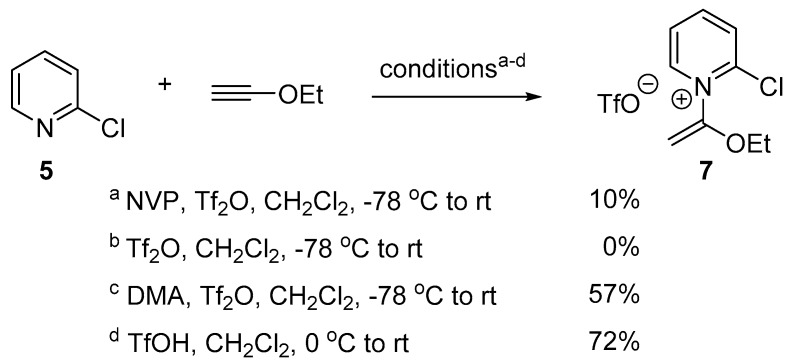
Optimized synthesis of 2-chloro-1-(1-ethoxyvinyl)pyridine-1-ium trifluoromethanesulfonate (**7**).

Since we became interested in studying the unique reactivity of *N*-(1-alkoxyvinyl)pyridinium salts en route to valuable heterocyclic products, we focused on optimizing the synthesis of these compounds. To begin, we first reinvestigated the role of NVP (**1**) in production of pyridinium salt **7** (Scheme 2). Notably, no formation of adduct **7** was observed in the absence of **1**, however, we were able to produce **7** in moderate yield when **1** was replaced with the simpler and less expensive tertiary amide *N*,*N*-dimethylacetamide (DMA) (Scheme 2, conditions a–c). This result suggested that the role of the amide in formation of **7** was to break down triflic anhydride into triflic acid adventitiously, allowing for acid-promoted formation of the highly electrophilic *O*-ethyl ketenium ion **4** (cf. Scheme 1). Accordingly, we were pleased to find that simple treatment of 2-chloropyridine (**5**) and ethoxyacetylene with triflic acid in a 1:1:1 molar ratio provides *N*-(1-ethoxyvinyl)pyridinium salt **7** in good yield and high purity, following chromatographic purification on silica gel (Scheme 2, condition d).

**Scheme 3:**
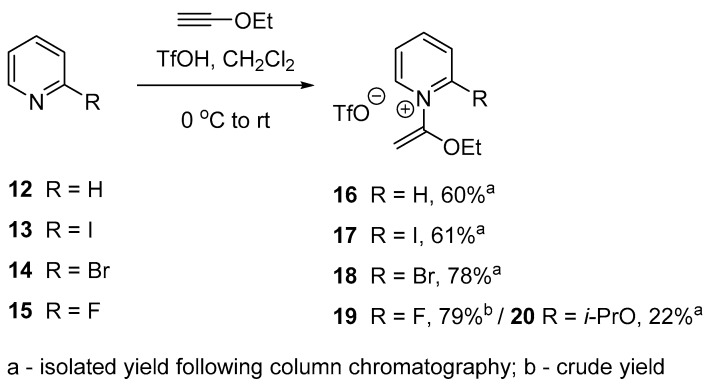
Synthesis of *N*-(1-ethoxyvinyl)pyridinium triflates with alternative pyridines.

This same protocol was applied to other 2-halopyridines **13**–**15**, producing adducts **17**–**19** (Scheme 3). Similarly, use of simple pyridine (**12**) delivers adduct **16** in moderate yield. Each *N*-(1-alkoxyvinyl)pyridinium salt product was found to be stable [49] upon purification via silica gel chromatography in isopropanol/chloroform, with the exception of the 2-fluoro analogue **19**, which converts rapidly to the isopropoxy S_N_Ar substitution product **20** under these conditions. Several attempts to replace isopropanol with non-nucleophilic polar solvents such as acetonitrile, acetone, and ethyl acetate were made, but without success. Nevertheless, a moderately pure sample of 2-fluoropyridinium salt **20** can be obtained by simply concentrating the reaction mixture in vacuo.

**Scheme 4:**
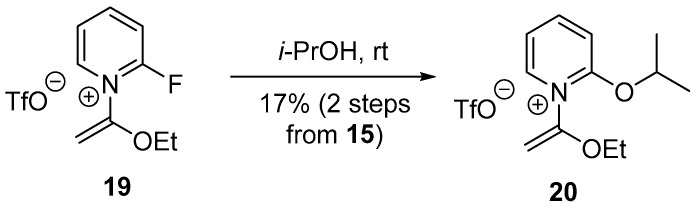
Nucleophilic aromatic substitution (S_N_Ar) of **19** with isopropanol.

Accordingly, when crude 2-fluoropyridinium salt **19** was stirred for 20 h in isopropanol, S_N_Ar product **20** is cleanly produced, albeit in low yield, presumably due, in part, to the facile decomposition of the starting material (Scheme 4). However, the ability of such compounds to partake in nucleophilic substitutions opens up a range of possibilities in which the S_N_Ar mechanism is operative. Further exploration of *N*-(1-alkoxyvinyl)pyridinium salts as synthons and reagents in organic synthesis will be reported in due course.

**Scheme 5:**
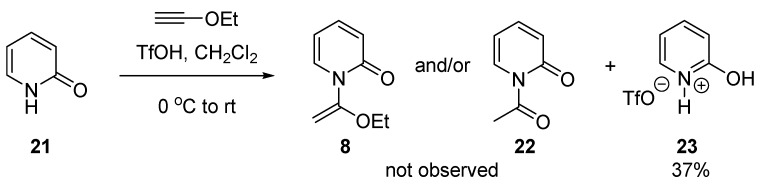
Attempted synthesis of pyridones **8** and **22**.

For comparison of our procedure with Weinstein’s protocol [38], we attempted to generate *N*-(1-ethoxyvinyl)-2-pyridone (**8**) and/or *N*-acylpyridone **22** (a hydrolysis side product observed in Weinstein’s reaction) from TfOH-promoted addition of ethoxyacetylene to 2-pyridone (**21**) (Scheme 5). However, only a mixture of unreacted **21** and the pyridinium triflate salt **23**, resulting from competitive *N*-protonation, were isolated.

### 2.3. Single Crystal X-ray Crystallography

Due to the novelty of the *N*-(1-alkoxyvinyl)pyridinium scaffold, we were prompted to obtain more extensive structural characterization through X-ray crystallography. In addition, we were motivated to gain insight into conformational preferences of such compounds within the solid state that may affect their stability and reactivity, such as the degree to which the π-orbitals of the vinyl group are conjugated to those within the pyridinium ring.

**Figure 2:**
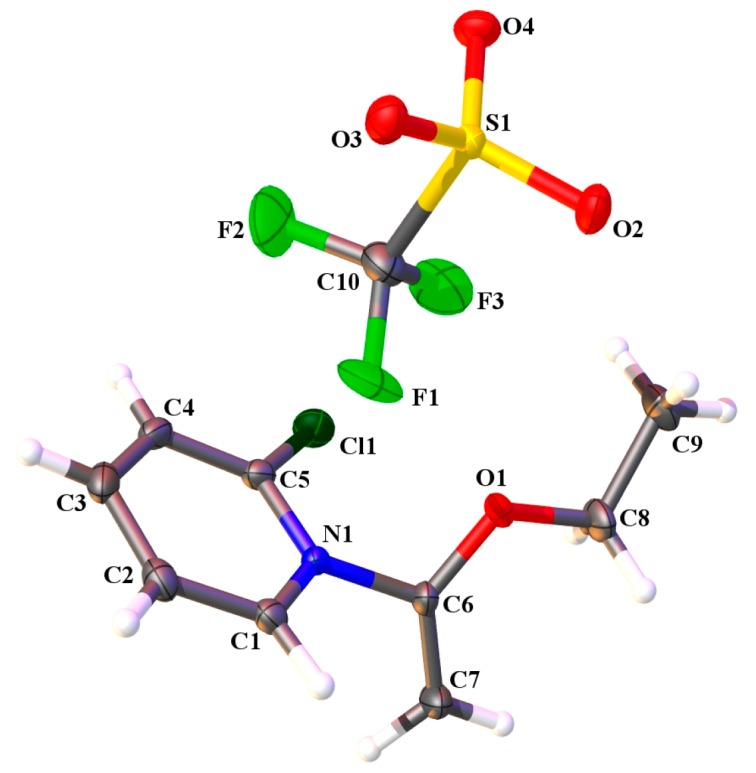
Crystal structure of **7** with anisotropic displacement ellipsoids set to 50% probability.

After multiple attempts, we were able to successfully grow crystals of *N*-(1-alkoxyvinyl)pyridinium salt **7** through slow evaporation of a concentrated solution in 1:1 chloroform/ethyl acetate (Figure 2). The crystallographic and refinement data for **7** is listed in Table 1.

**Figure 3:**
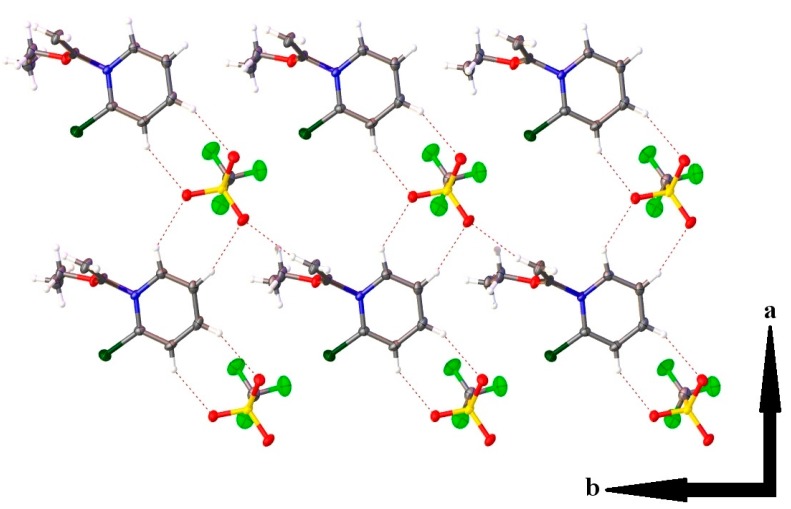
Packing of **7** relative to the *ab*-plane. Weak hydrogen bonds involving the three oxygen atoms of the –SO_3_ group and the hydrogen atoms from the aromatic rings of the pyridinium cation are represented by dashed lines.

The solid state structure of **7** crystallized in the centrosymmetric triclinic space group *P-1*, and had one pyridinium cation and triflate anion as the elements of the asymmetric unit. Within the *ab*-plane, the sulfonate groups from the [CF_3_SO_3_]^−^ anions and the aromatic rings from pyridinium cations are oriented in a checkerboard pattern, extending infinitely along both the *a*- and *b*-axes (Figure 3). No evidence of [CF_3_SO_3_]^−^ disorder was observed. Sandwiched between the layers containing the –SO_3_ groups and the aromatic rings of pyridinium cations, relative to the *c*-axis, is a layer encompassing –CF_3_ and ethoxy groups from symmetry-equivalent triflate anions and pyridinium cations. The ethoxy groups from all pyridinium cations propagate inward in a manner analogous to that of long-chain saturated hydrocarbon chains seen within lipid bilayers. No evidence for the presence of supramolecular interactions involving these moieties within that latter layer was observed.

**Figure 4:**
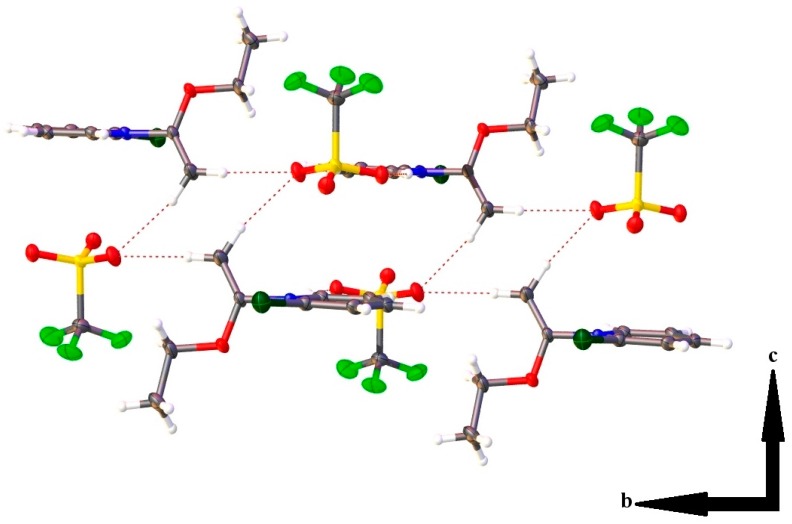
Packing of **7** relative to the *bc*-plane. Four-component assemblies of weak hydrogen bonds, encompassing vinylic hydrogen and sulfonate oxygen atoms, are represented by dashed lines.

Unlike the –CF_3_/ethoxy layer, within the layer encompassing the aromatic rings of pyridinium cations and –SO_3_ groups, all three oxygen atoms of the sulfonate group engage in weak hydrogen bonding (Figure 3) [50]. Upon closer analysis, the three oxygen atoms from the various symmetry equivalent triflate anions within the checkerboard layer form weak hydrogen bonds with hydrogen atoms bound to *sp*^2^-hybridized carbon atoms from the aromatic rings of neighboring pyridinium cations. While –CF_3_ and ethoxy groups are sandwiched between layers of aromatic rings and –SO_3_ groups, a repeat pattern of this bilayer-like assembly mirrors the *c*-axis, allowing layers containing aromatic rings and –SO_3_ groups to come in contact with one another. As a direct consequence of this contact, a four-component construct encompassing weak hydrogen bonds between two vinyl groups and two oxygen atoms from symmetry equivalent [CF_3_SO_3_]^−^ anions results (Figure 4). A table summarizing distances, angles, and symmetry operations for all hydrogen bonding interactions within the solid state structure of **7** is included in Table 2.

### 2.4. Computational Modeling

For further insight into conformational preferences and rotational dynamics of *N*-(1-alkoxyvinyl)pyridinium salts, we calculated the energy profile of the rotation around the exocyclic C–N bond of both **7** and the less sterically constrained **16**, using density functional theory (see Section 3.4 for details). Since the π-bond of the exocyclic vinyl group within these systems can overlap with the aromatic π-system, we hypothesize that the rotational conformation of this group with respect to the pyridinium ring will have a significant impact on their overall stability and reactivity. The calculations are able to both predict the lowest energy conformations and the barriers to rotation, and thus, the degree to which a planar conformation is achieved.

**Figure 5:**
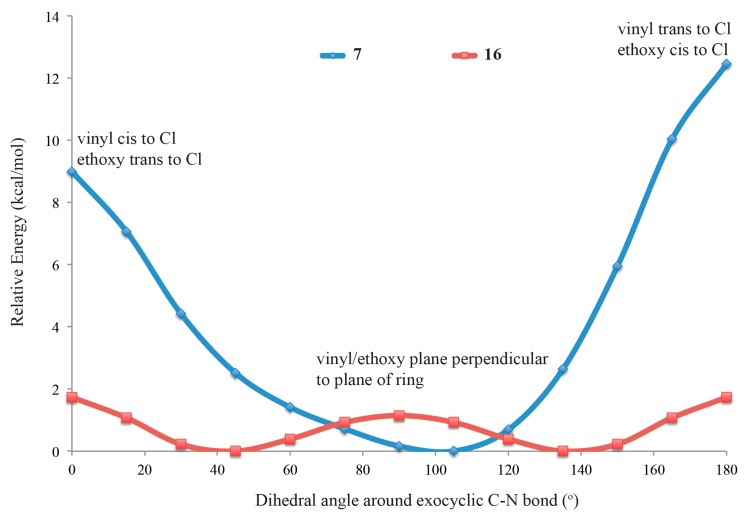
Energy profile of the rotation of the ethoxy and vinyl groups around the exocyclic C–N bond. The profile for compound **7** is shown in blue, and the profile for compound **16** is shown in red. Note the profile of compound **7** is symmetric around 0° and 180°, while the profile of compound **16** is symmetric around 0°, 90°, 180°, and 270°.

The steric bulk of the chlorine on the ring of **7** strongly dictates the rotational conformation of the vinyl and ethoxy groups. As shown in Figure 5, the predicted minimum energy conformation of the dihedral angle around the exocyclic C–N bond is approximately 105°, very near to a perpendicular (90°) orientation of these groups relative to the plane of the ring that minimizes steric interactions. The calculated barriers to rotation that bring either the vinyl or ethoxy groups into close contact with the ring chlorine are consistent with this interpretation and predicts that the molecule will not often be in a conformation where the π-systems of the ring and vinyl groups can overlap. In a conformation with the vinyl group cis to chlorine, the calculated energy is approximately 9 kcal/mol, while when the ethoxy group is cis to the chlorine, the calculated energy is slightly greater than 12 kcal/mol.

Compound **16**, which has the chlorine replaced with a hydrogen, exhibits much lower steric constraints to rotation, however. The calculated minimum energy for the exocyclic C–N bond dihedral angle is 135° (equivalent to 45°), which is much closer to planar configuration (180°) than the 105° of compound **7**. In fact, compound **16** exhibits a local maximum in its energy profile at 90°, as this orientation completely disallows any conjugation of the vinyl group’s π-bond with the ring’s aromatic π-system. The barrier to the fully planar conformation is much less in this compound, and is less than 2 kcal/mol, making this a much easier conformation to realize.

**Figure 6:**
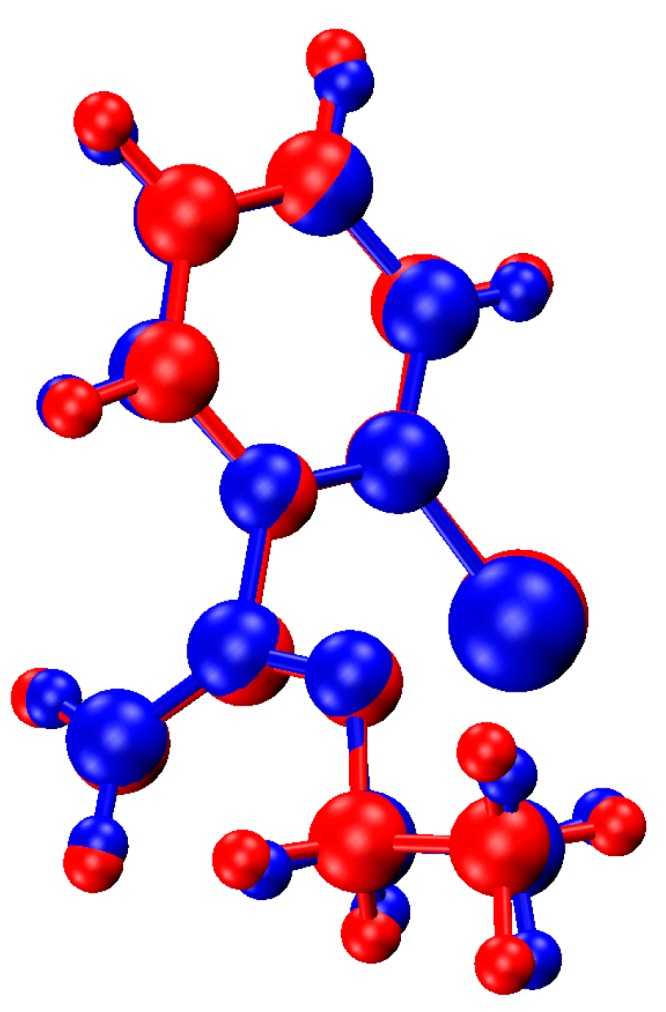
Overlay of crystal structure (blue) and predicted global energy minimum from density functional theory calculations (red) of compound **7**.

While the absolute barrier heights and position of the extrema are approximate, we are relatively confident in the predicated energy profiles of these compounds because the predicted global energy minimum structure of compound **7** matches very closely with the experimentally determined X-ray crystal structure. The two structures are overlaid in Figure 6, and have a heavy-atom RMSD of only 0.118 Å.

## 3. Materials and Methods

### 3.1. General Experimental Methods

All chemicals were purchased from Sigma-Aldrich, St. Louis, MO, USA, and used without further purification. Column chromatography was performed using 4 g RediSep Rf Gold Normal Phase Silica Columns (20–40 micron) with a Teledyne Isco CombiFlash Rf200 purification system. NMR Spectra were obtained on a Bruker Avance 500 MHz NMR. Both nominal and high-resolution mass spectra were obtained on a Waters Micromass 70-VSE. For all pyridinium salts, only the pyridinium cation was detected and analyzed by mass spectrometry, due to weak coordination by the triflate anion. Melting points were determined using a SRS DigiMelt MPA160. X-ray data was collected with a Rigaku Oxford Diffraction Synergy-S single crystal X-ray diffractometer equipped with a Cu PhotonJet micro-focus source and Pilatus P200K HPC detector.

### 3.2. Synthesis of N-(1-Ethoxyvinyl)pyridinium Triflates

General procedure for the synthesis of *N*-(1-ethoxyvinyl)pyridinium triflates (Method A): Trifluoromethanesulfonic acid (179 μL, 2 mmol) was slowly added to a stirred solution of requisite pyridine (2 mmol), ethoxyacetylene solution (479 μL, 2 mmol, ~40 wt % in hexanes), and dichloromethane (3.5 mL) at 0 °C. The resulting solution was stirred for 5 min at this temperature, before removing the ice bath and stirring for 18 h, gradually warming to room temperature. The resulting dark reddish-brown reaction mixture was concentrated in vacuo to provide a crude residue, which was purified by silica gel column chromatography with a 0–100% chloroform/isopropanol gradient, beginning with pure chloroform to elute nonpolar impurities and ending with pure isopropanol.

#### 3.2.1. Synthesis of 2-Chloro-1-(1-ethoxyvinyl)pyridine-1-ium Trifluoromethanesulfonate (**7**)

Using Method A with 2-chloropyridine (176 μL, 2 mmol), compound **7** was obtained as an amorphous white solid (481 mg, 72%). ^1^H-NMR (500 MHz, D_2_O) δ 8.99 (dd, *J* = 6.2, 1.8 Hz, 1H), 8.64 (td, *J* = 8.1, 1.8 Hz, 1H), 8.24 (dd, *J* = 8.4, 1.2 Hz, 1H), 8.06 (ddd, *J* = 7.7, 6.2, 1.3 Hz, 1H), 4.88 (d, *J* = 5.3 Hz, 1H), 4.76 (d, *J* = 5.4 Hz, 1H), 4.20 (q, *J* = 7.0 Hz, 2H), 1.36 (t, *J* = 7.1 Hz, 3H); ^13^C-NMR (125 MHz, D_2_O) δ 153.1, 149.7, 147.3, 147.1, 130.1, 126.2, 119.7 (q, ^1^*J_CF_* = 315 Hz, *C*F_3_), 87.2, 67.8, 13.2; LRMS-ES+ *m*/*z* (relative intensity) 184.1 (C_9_H_11_NOCl M+, 38 (Cl-35 isotope)), 186.1 (C_9_H_11_NOCl M+, 14 (Cl-37 isotope)); HRMS-ES+ (C_9_H_11_NOCl) calcd. 184.0529 (M+), found 184.0532; m.p.: 101–103 °C.

Method B (with Tf_2_O and NVP; Scheme 2a): Trifluoromethanesulfonic anhydride (167 μL, 1.0 mmol) was slowly added to a stirred solution of 2-chloropyridine (170 μL, 1.8 mmol), *N*-vinyl 2-pyrrolidinone (100 mg, 0.9 mmol), and dichloromethane (3 mL) at −78 °C, and the resulting solution was stirred for 5 min. Following this, the reaction vessel was transferred to a 0 °C ice bath, and stirred for an additional 2 min before adding ethoxyacetylene solution (430 μL, 1.8 mmol, ~40 wt % in hexanes). After stirring the resulting solution for 5 min at 0 °C, the ice bath was removed, and the reaction was stirred for 1 h, gradually warming to room temperature. The resulting dark red reaction mixture was concentrated in vacuo to provide a crude residue, which was purified by silica gel column chromatography (0–100% chloroform/isopropanol gradient on a 4 g RediSep Rf Gold cartridge). Compound **7** eluted from the pure isopropanol fractions to yield an amorphous light brown solid (33 mg, 10%). Recrystallization and slow evaporation of the product in 1:1 chloroform/ethyl acetate provided pale yellow block-like crystals that were used for X-ray analysis.

Method C (with Tf_2_O and DMA; Scheme 2c): The same protocol as Method B was followed on a 0.9 mmol scale, except that *N*-vinyl 2-pyrrolidinone was replaced with *N*,*N*-dimethylacetamide (84 μL, 0.9 mmol). Compound **7** was isolated as a pale yellow solid (171 mg, 57%).

#### 3.2.2. Synthesis of 1-(1-Ethoxyvinyl)pyridine-1-ium Trifluoromethanesulfonate (**16**)

Using Method A with pyridine (162 μL, 2 mmol), compound **16** was obtained as a yellow residue (359 mg, 60%). ^1^H-NMR (500 MHz, D_2_O) δ 9.12–9.04 (m, 2H), 8.66 (tt, *J* = 7.9, 1.4 Hz, 1H), 8.11 (app t, *J* = 6.8 Hz, 2H), 4.89 (d, *J* = 5.7 Hz, 1H), 4.69 (app d overlapping with HDO signal, *J* = 6.2 Hz, 1H), 4.21 (q, *J* = 7.0 Hz, 2H), 1.42 (t, *J* = 7.0 Hz, 3H); ^13^C-NMR (125 MHz, D_2_O) δ 154.6, 148.3, 142.1, 127.7, 119.7 (q, ^1^*J_CF_* = 315 Hz, *C*F_3_), 83.1, 67.9, 13.2; LRMS-ES+ *m*/*z* (relative intensity) 150.1 (C_9_H_12_NO M+, 10); HRMS-ES+ (C_9_H_12_NO) calcd. 150.0919 (M+), found 150.0926.

#### 3.2.3. Synthesis of 2-Iodo-1-(1-ethoxyvinyl)pyridine-1-ium Trifluoromethanesulfonate (**17**)

Using Method A with 2-iodopyridine (217 μL, 2 mmol), compound **17** was obtained as an amorphous pale yellow solid (519 mg, 61%). ^1^H-NMR (500 MHz, CDCl_3_) δ 9.18 (dd, *J* = 6.0, 1.8 Hz, 1H), 8.59 (dd, *J* = 8.0, 1.5 Hz, 1H), 8.30 (td, *J* = 8.0, 1.8 Hz, 1H), 8.24 (ddd, *J* = 7.6, 6.0, 1.5 Hz, 1H), 4.89 (d, *J* = 5.3 Hz, 1H), 4.71 (d, *J* = 5.4 Hz, 1H), 4.17 (q, *J* = 7.0 Hz, 2H), 1.45 (t, *J* = 7.0 Hz, 3H); ^13^C-NMR (125 MHz, CDCl_3_) δ 157.1, 148.6, 147.1, 141.5, 128.2, 120.7 (q, ^1^*J_CF_* = 319 Hz, *C*F_3_), 114.2, 87.2, 67.5, 13.9; LRMS-ES+ *m*/*z* (relative intensity) 276.0 (C_9_H_11_NOI M+, 48); HRMS-ES+ (C_9_H_11_NOI) calcd. 275.9885 (M+), found 275.9888; m.p.: 93–98 °C.

#### 3.2.4. Synthesis of 2-Bromo-1-(1-ethoxyvinyl)pyridine-1-ium Trifluoromethanesulfonate (**18**)

Using Method A with 2-bromopyridine (193 μL, 2 mmol), compound **18** was obtained as an amorphous off-white solid (590 mg, 78%). ^1^H-NMR (500 MHz, CDCl_3_) δ 9.14 (dd, *J* = 6.2, 1.7 Hz, 1H), 8.61 (td, *J* = 8.0, 1.7 Hz, 1H), 8.34 (dd, *J* = 8.2, 1.3 Hz, 1H), 8.28 (ddd, *J* = 7.6, 6.0, 1.3 Hz, 1H), 4.94 (d, *J* = 5.4 Hz, 1H), 4.75 (d, *J* = 5.4 Hz, 1H), 4.17 (q, *J* = 7.0 Hz, 2H), 1.43 (t, *J* = 7.0 Hz, 3H); ^13^C-NMR (125 MHz, CDCl_3_) δ 154.6, 149.2, 148.8, 137.1, 134.1, 127.9, 120.6 (q, ^1^*J_CF_* = 318 Hz, *C*F_3_), 87.5, 67.5, 13.8; LRMS-ES+ *m*/*z* (relative intensity) 228.0 (C_9_H_11_NOBr M+, 37 (Br-79 isotope)), 230.0 (C_9_H_11_NOBr M+, 35 (Br-81 isotope)); HRMS-ES+ (C_9_H_11_NOBr) calcd. 228.0024 (M+), found 228.0032; m.p.: 111–115 °C.

#### 3.2.5. Synthesis of 2-Fluoro-1-(1-ethoxyvinyl)pyridine-1-ium Trifluoromethanesulfonate (**19**)

Using Method A with 2-fluoropyridine (172 μL, 2 mmol) and without column chromatography, crude compound **19** was obtained as a brown solid (501 mg, ~79%). ^1^H-NMR (500 MHz, CDCl_3_) δ 8.86 (dddd, *J* = 8.7, 7.6, 5.7, 1.9 Hz, 1H), 8.77 (ddd, *J* = 6.0, 4.0, 1.9 Hz, 1H), 8.05 (ddd, *J* = 7.6, 6.2, 1.2 Hz, 1H), 7.93–7.87 (m, 1H), 4.99 (d, *J* = 5.5 Hz, 1H), 4.76 (d, *J* = 5.5 Hz, 1H), 4.13 (q, *J* = 7.0 Hz, 2H), 1.38 (t, *J* = 7.0 Hz, 3H); ^13^C-NMR (125 MHz, CDCl_3_) δ 157.4 (d, ^1^*J_CF_* = 284 Hz), 154.3 (d, ^3^*J_CF_* = 12.5 Hz), 149.3, 143.8 (d, ^3^*J_CF_* = 8.8 Hz), 125.0 (d, ^4^*J_CF_* = 3.8 Hz), 120.6 (q, ^1^*J_CF_* = 318 Hz, *C*F_3_), 115.1 (d, ^2^*J_CF_* = 18.8 Hz), 87.7, 67.8, 13.7; analysis of compound 19 by LRMS/HRMS and melting point was unsuccessful due to rapid decomposition.

#### 3.2.6. Synthesis of 1-(1-Ethoxyvinyl)-2-isopropoxypyridin-1-ium Trifluoromethanesulfonate (**20**)

Using Method A with 2-fluoropyridine (172 μL, 2 mmol), compound **20** was obtained as an amorphous off-white solid (157 mg, 22%). ^1^H-NMR (500 MHz, CDCl_3_) δ 8.55 (ddd, *J* = 9.2, 7.4, 1.9 Hz, 1H), 8.31 (ddd, *J* = 6.5, 1.9, 0.6 Hz, 1H), 7.86–7.79 (m, 1H), 7.53 (ddd, *J* = 7.4, 6.3, 1.1 Hz, 1H), 5.31 (hept, *J* = 6.1 Hz, 1H), 4.62 (d, *J* = 5.0 Hz, 1H), 4.58 (d, *J* = 4.9 Hz, 1H), 4.06 (q, *J* = 7.0 Hz, 2H), 1.48 (d, *J* = 6.1 Hz, 6H), 1.37 (t, *J* = 7.0 Hz, 3H); ^13^C-NMR (125 MHz, CDCl_3_) δ 158.8, 151.7, 150.8, 142.1, 120.8 (q, ^1^*J_CF_* = 319 Hz, *C*F_3_), 118.6, 113.1, 86.2, 79.3, 66.8, 21.4, 13.8; LRMS-ES+ *m*/*z* (relative intensity) 208.1 (C_12_H_18_NO_2_ M+, 15); HRMS-ES+ (C_12_H_18_NO_2_) calcd. 208.1338 (M+), found 208.1336; m.p.: 55–58 °C.

Method D (Two-step protocol; Scheme 4). Freshly prepared 2-fluoro-1-(1-ethoxyvinyl)pyridine-1-ium (**19**) (592 mg, 1.87 mmol) was dissolved in isopropanol (5 mL) and stirred for 18 h at room temperature. The resulting solution was concentrated in vacuo to provide a crude residue, which was purified by silica gel column chromatography with a 0–100% chloroform/isopropanol gradient, beginning with pure chloroform to elute nonpolar impurities, and ending with pure isopropanol. Compound **20** was obtained as an amorphous off-white solid (124 mg, 17% from **15**).

#### 3.2.7. Synthesis of 2-Hydroxypyridin-1-ium Trifluoromethanesulfonate (**23**)

Using Method A with 2-hydroxypyridine (190 mg, 2 mmol), side product **23** was obtained as an amorphous white solid (180 mg, 37%). ^1^H-NMR (500 MHz, CDCl_3_) δ 8.02 (ddd, *J* = 9.0, 7.1, 1.9 Hz, 1H), 7.96 (ddd, *J* = 6.4, 2.0, 0.8 Hz, 1H), 7.14 (dt, *J* = 9.0, 0.9 Hz, 1H), 7.04 (ddd, *J* = 7.2, 6.3, 1.1 Hz, 1H); ^13^C-NMR (125 MHz, CDCl_3_) δ 161.9, 146.4, 136.4, 120.0 (q, ^1^*J_CF_* = 317 Hz, *C*F_3_), 116.6, 114.6; LRMS-ES+ *m*/*z* (relative intensity) 96.0 (C_5_H_6_NO M+, 40); HRMS-ES+ (C_5_H_6_NO) calcd. 96.0449 (M+), found 96.0448; m.p.: 143–149 °C.

### 3.3. X-ray Diffraction Methodology

A pale yellow block-like crystal of 7 having dimensions 0.29 × 0.32 × 0.40 mm^3^ was secured to a cryoloop using Paratone oil. The single crystal reflection data was collected on a Rigaku Oxford Diffraction Synergy-S X-ray diffractometer equipped with a Pilatus P200K hybrid photon counting (HPC) detector. The data were collected at 100 K using Cu K_α1_ radiation from a data collection strategy calculated using CrysAlis^Pro^, which was also responsible for unit cell determination, initial indexing, data collection, frame integration, Lorentz-polarization corrections, and final cell parameter calculations [51]. Multi-scan absorption corrections were performed using the SCALE3 ABSPACK algorithm integrated into CrysAlis^Pro^ [52]. The crystal structure was solved via intrinsic phasing using ShelXT and refined using ShelXL within the Olex2 graphical user interface [53,54]. The structural model’s space group was unambiguously verified by PLATON [55]. The final structural refinement included anisotropic temperature factors on all non-hydrogen atoms and hydrogen atoms were attached via the riding model at calculated positions using appropriate HFIX commands.

### 3.4. Computational Methodology

All calculations were carried out using Gaussian 03, Revision D.01 [56] using the density functional theory method [57] with the B3LYP functional [58,59]. Geometry optimizations were carried out with the exocyclic C–N bond dihedral angle fixed at 15° intervals, from 0° to 180° for compound **7**, and from 0° to 90° for compound **16**. Compound **7** is symmetric around 0° and 180°; compound **16** is symmetric around 0°, 90°, 180°, and 270°. Several local minima of the ethoxy group dihedral angles were observed so optimizations from several different starting geometries were initiated to confirm that the expected trans configurations were indeed the lowest energy conformations. The cc-pvdz basis set was used for geometry optimizations, and subsequent single point calculations from the optimized geometries utilized the cc-pvqz basis set [60].

## 4. Conclusions

We have shown that several *N*-(1-ethoxyvinyl)pyridinium triflates can be prepared in a straightforward manner from ethoxyacetylene, pyridines, and triflic acid. We anticipate that the ease of synthesis and general benchtop stability of these unusual *N*-quaternized ketene *N*,*O*-acetals will enable their use as reagents in organic synthesis. Further evaluation of the substrate scope of this reaction, improved purification methods, and general reactivity of *N*-(1-alkoxyvinyl)pyridinium salts is currently underway in our laboratory.

Concurrent structural analysis via single crystal X-ray diffraction and computational methods, successfully and independently confirmed the lowest energy conformation of 2-chloro-1-(1-ethoxyvinyl)pyridine-1-ium trifluoromethanesulfonate (**7**). Computational modeling of **7** predicted that the compound was unlikely to be stabilized by interactions between the vinyl group and the ring’s aromatic system. Such information will be valuable as further studies on the reactivity of this novel compound class are pursued.

## Figures and Tables

**Table 1:** Crystallographic and refinement data for 2-chloro-1-(1-ethoxyvinyl)pyridine-1-ium trifluoromethanesulfonate (**7**).

Compound	7
CCDC Code	1815230
Formula	C_10_H_11_ClF_3_NO_4_S
Formula weight	333.71
Temp.	100(2)
Space group	*P-1*
a, Å	8.5347(2)
b, Å	9.0911(2)
c, Å	9.2950(2)
α, deg	85.871(2)
β, deg	80.449(2)
γ, deg	82.656(2)
Volume, Å^3^	704.41(3)
Z	2
Density (calculated), mg/m^3^	1.573
μ, mm^−1^	4.259
Scan	*ω* scan
θ range for data collection, deg	6.582–67.716
Reflections measured	5893
Independent observed reflns.	2522
Independent reflns. [*I* > 2σ]	2467
Data/restraints/parameters	2522/0/217
*R*_int_	0.0194
Final *R* Indices [*I* > 2σ]	*R*_1_ = 0.0322,
	*wR*2 = 0.0858
*R* Indices (all data)	*R*_1_ = 0.0329,
	*wR*2 = 0.0864
Goodness-of-fit on *F*^2^	1.085

**Table 2:** Distances and symmetry operations of hydrogen bonding interactions within the solid state structure of **7**.

Hydrogen Bonding Interaction	H•••O/C•••O Bond Distances (Å)	C–H•••O Bond Angle (°)	Symmetry Operation
C(1)–H(1)•••O(2)	2.37(2)/3.139(2)	134.0(16)	-x, 2-y, 1-z
C(2)–H(2)•••O(4)	2.35(2)/3.268(2)	163.5(18)	-x, 2-y, 1-z
C(3)–H(3)•••O(3)	2.41(2)/3.276(2)	156.8(18)	1-x, 2-y, 1-z
C(4)–H(4)•••O(2)	2.46(2)/3.259(2)	144.5(17)	1-x, 2-y, 1-z
C(7)–H(7A)•••O(4)	2.46(2)/3.390(2)	156.6(17)	x, y, z-1
C(7)–H(7B)•••O(4)	2.42(3)/3.350(2)	168.1(19)	-x, 1-y, 1-z

## Supplementary Materials

Crystallographic data for **7** has been deposited with the Cambridge Crystallography Date Center, 12 Union Road, Cambridge CB22 1EZ, UK (Fax: +44-1223-336-033; E-mail: deposit@ccdc.ca-m.ac.uk or http://www.ccdc.cam.ac.uk) and are available freely on request quoting the deposition number CCDC-1815230. ^1^H- and ^13^C-NMR spectra of *N*-(1-ethoxyvinyl)pyridinium triflates **7**, **14**–**20**, and **23** and crystal data files of **7** are provided as supplementary data.
