# Supplementary material for: Synthesis, Characterization, and Computational Modeling of N-(1-Ethoxyvinyl)pyridinium Triflates, an Unusual Class of Pyridinium Salts
Source: Molecules. 2018 Feb 14;23(2):413. doi: 10.3390/molecules23020413 (PMC6017145; doi:10.3390/molecules23020413)
Supplement: Supplementary file 1 [file molecules-23-00413-s001.zip › crystal files for 7/Supplementary Materials.pdf]

Supplementary Materials: Synthesis, Characterization, and Computational Modelling of *N*-(1-Ethoxyvinyl)pyridinium Triflates, an Unusual Class of Pyridinium Salts

Jonathan D. Shapiro[1], Justin C. Sonberg[1], Benjamin C. Schafer[1], Christopher C. Williams[1], Hannah R. Ferris[1], Eric W. Reinheimer[2], Adam W. Van Wynsberghe[1], Charles E. Kriley[3] and Max M. Majireck[1,*]

[1]Department of Chemistry, Hamilton College, 198 College Hill Road, Clinton, NY, 13323, USA; Email: mmajirec@hamilton.edu

[2]Rigaku Oxford Diffraction, 9009 New Trails Drive, The Woodlands, TX, 77381, USA

[3]Department of Chemistry, Grove City College, 100 Campus Drive, Grove City, PA, 16127, USA

*Correspondence: mmajirec@hamilton.edu; Tel.: +001-315-859-4742

Contents:

^{1}H and ^{13}C NMR Spectra of Key Compounds…………………………………….............. S2- S15
2-Chloro-1-(1-ethoxyvinyl)pyridine-1-ium trifluoromethanesulfonate (**7**)…...…..... S2- S3
1-(1-Ethoxyvinyl)pyridine-1-ium trifluoromethanesulfonate (**16**)……………...….. S4- S5
2-Iodo-1-(1-ethoxyvinyl)pyridine-1-ium trifluoromethanesulfonate (**17**)……….…... S6- S7
2-Bromo-1-(1-ethoxyvinyl)pyridine-1-ium trifluoromethanesulfonate (**18**)……..….. S8- S9
2-Fluoro-1-(1-ethoxyvinyl)pyridine-1-ium trifluoromethanesulfonate (**19**)…....…. S10- S11
1-(1-Ethoxyvinyl)-2-isopropoxypyridin-1-ium trifluoromethanesulfonate (**20**).... S12- S13
2-Hydroxypyridin-1-ium trifluoromethanesulfonate (**23**)………………………... S14-S15

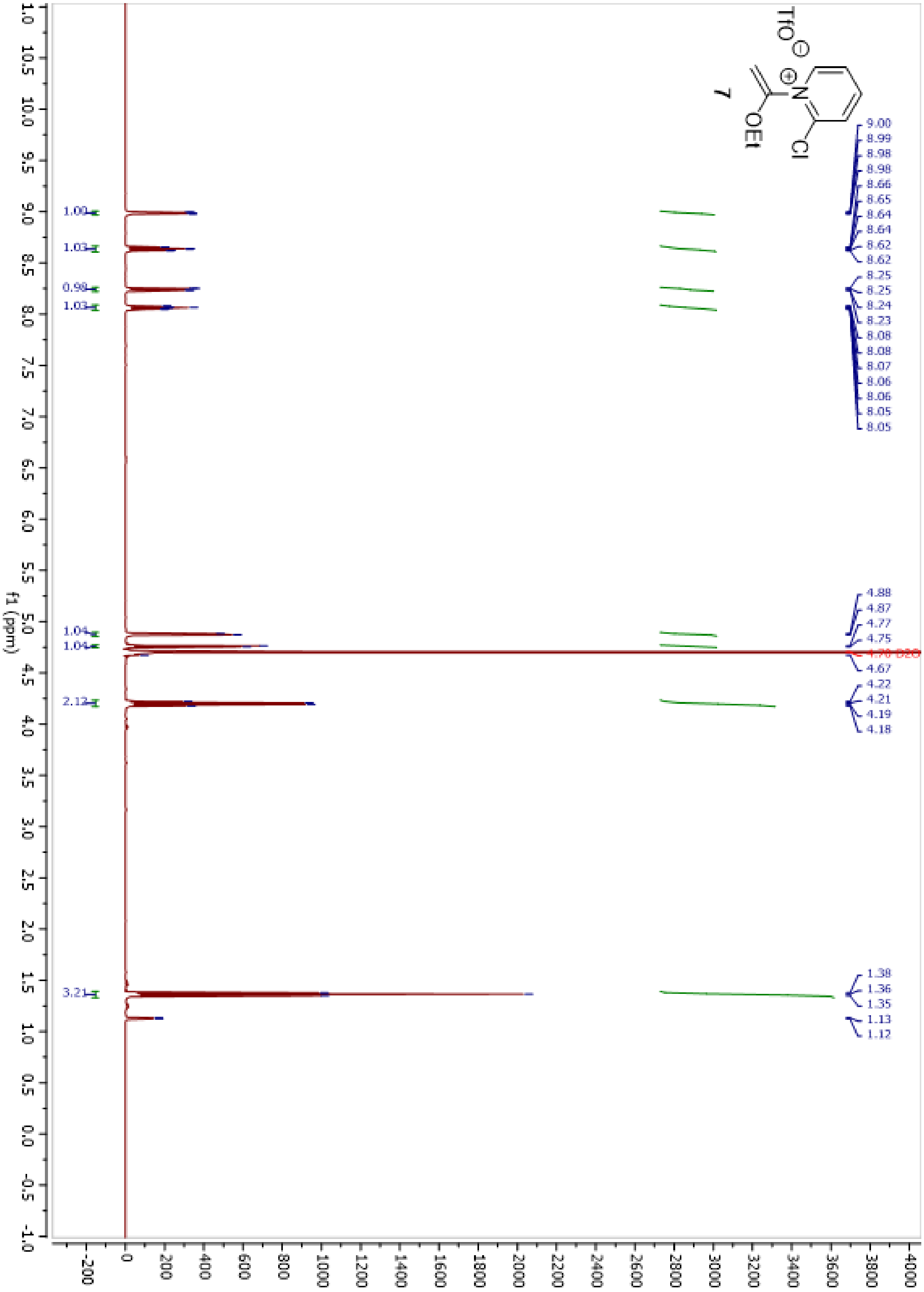
TfO
Cl
OEt
7
f1 (ppm)
4.70 D2O

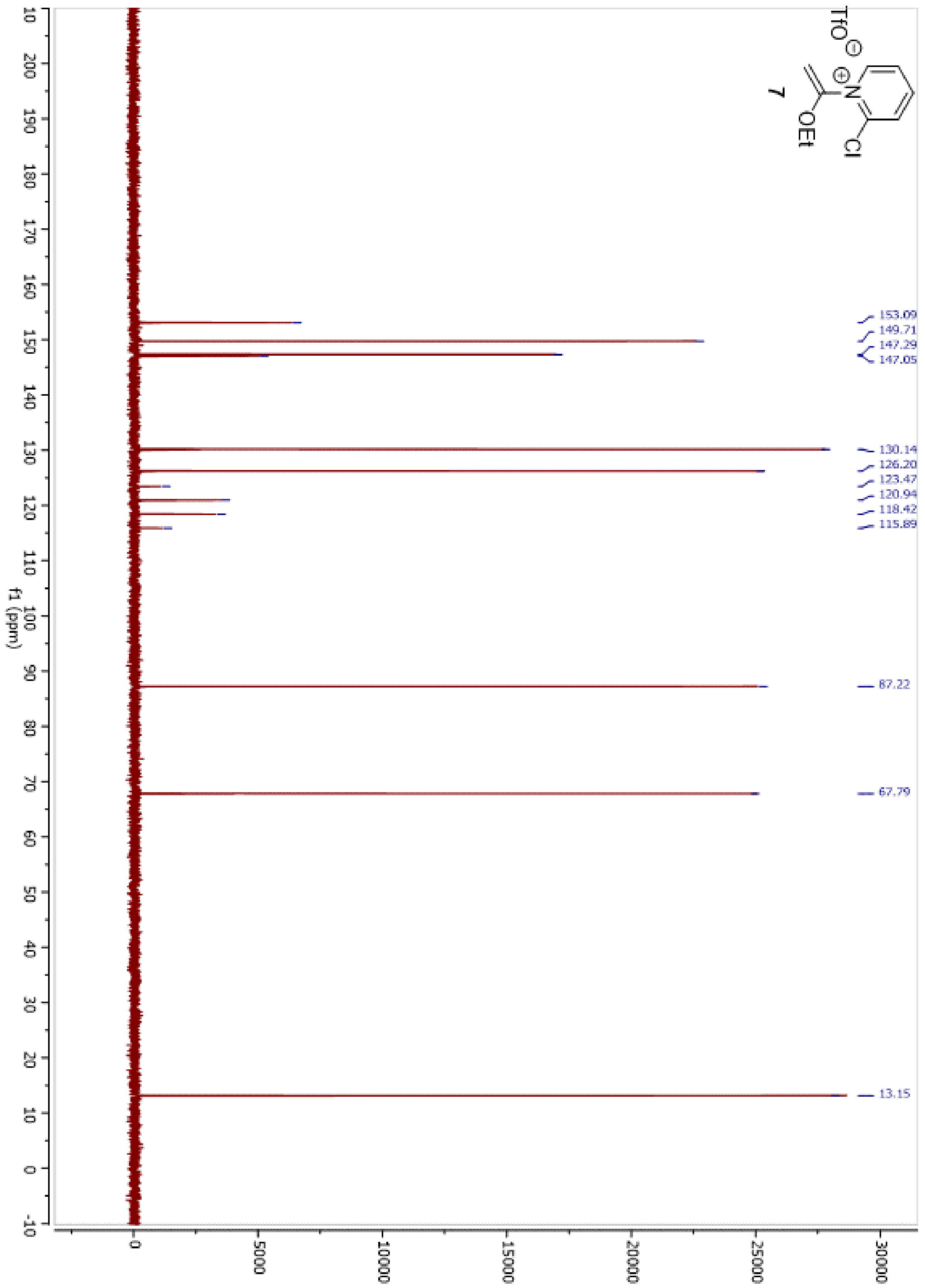

TfO
N
Cl
OEt
7
153.09
149.71
147.29
147.05
130.14
126.20
123.47
120.94
118.42
115.89
87.22
67.79
13.15
f1 (ppm)

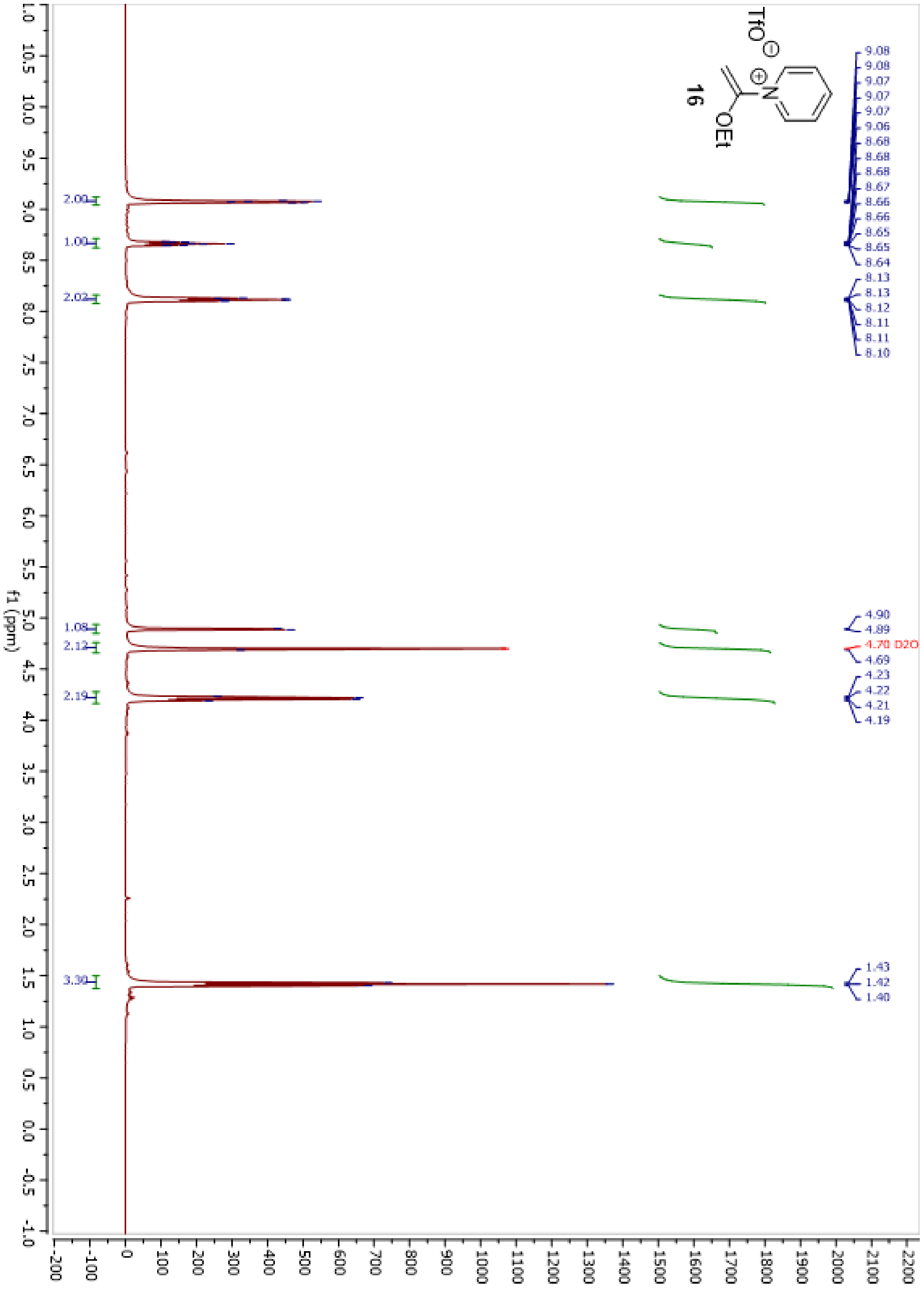

TfO
OEt
16
9.08
9.08
9.07
9.07
9.07
9.06
8.68
8.68
8.68
8.67
8.66
8.66
8.65
8.65
8.64
8.13
8.13
8.12
8.11
8.11
8.10
4.90
4.89
4.70 D2O
4.69
4.23
4.22
4.21
4.19
1.43
1.42
1.40
2.00
1.00
2.02
1.08
2.12
2.19
3.30
f1 (ppm)

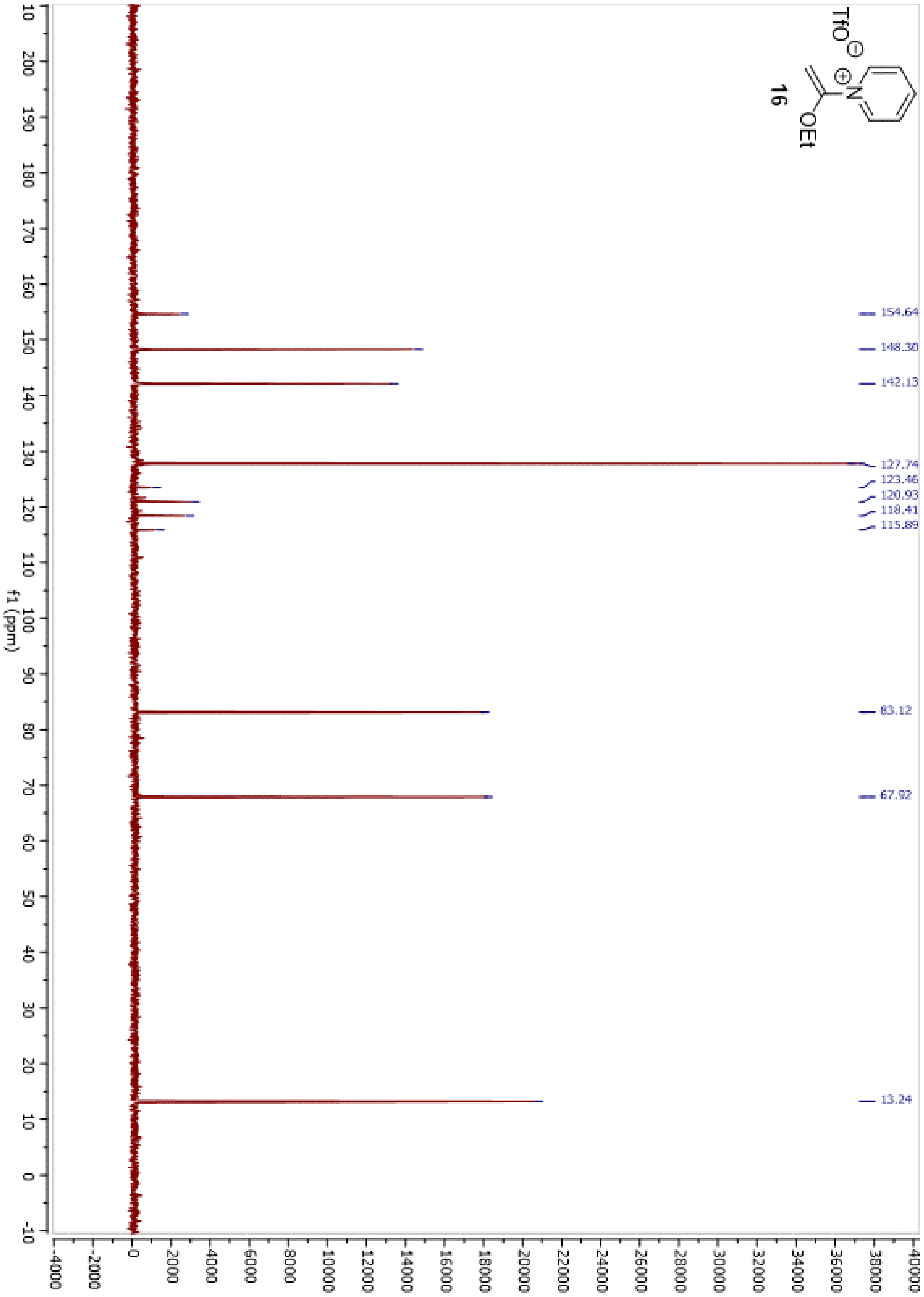
TfO
16
OEt
154.64
148.30
142.13
127.74
123.46
120.93
118.41
115.89
83.12
67.92
13.24
f1 (ppm)

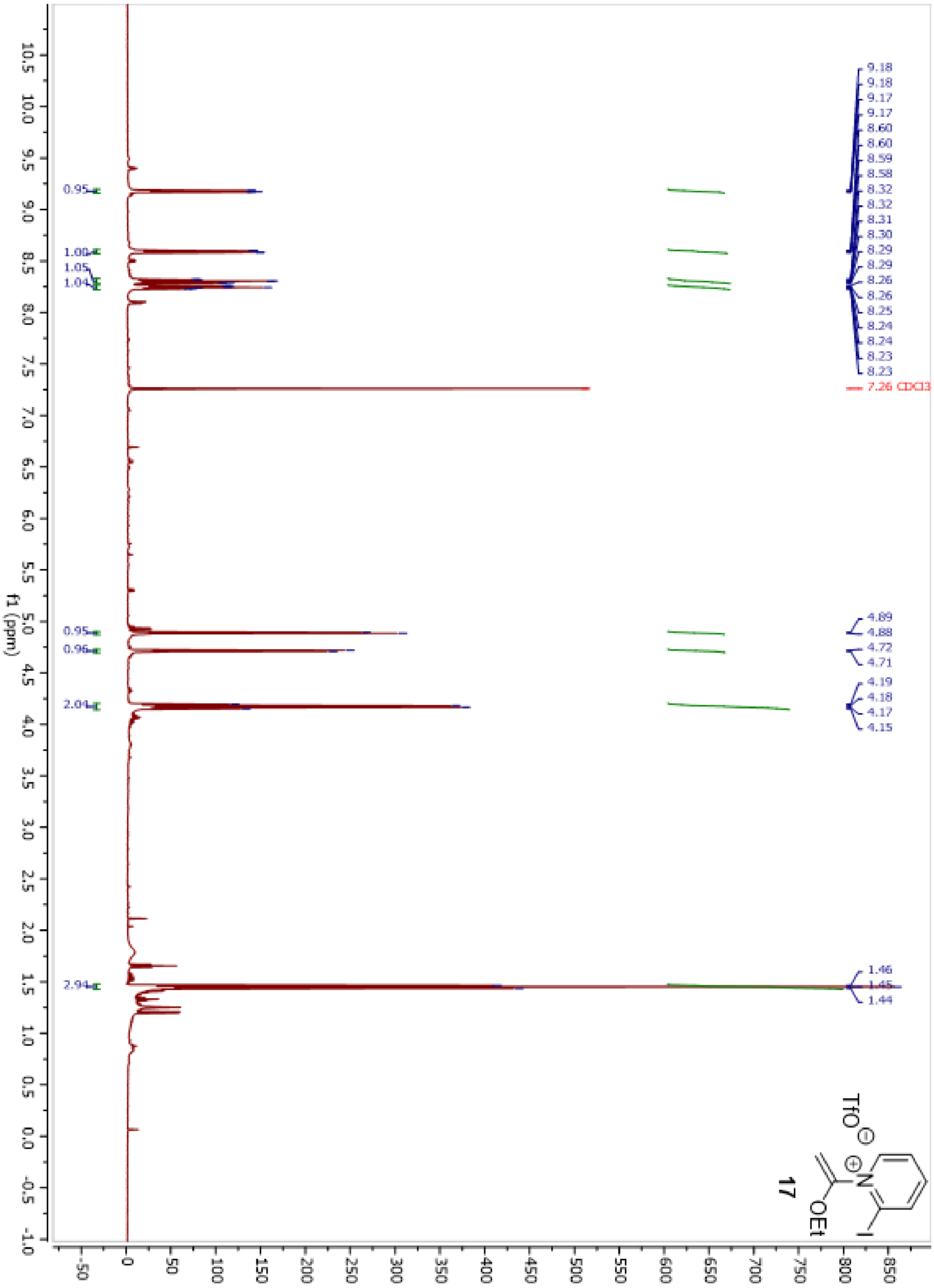
9.18
9.18
9.17
9.17
8.60
8.60
8.59
8.58
8.32
8.32
8.31
8.30
8.29
8.29
8.26
8.26
8.25
8.24
8.24
8.23
8.23
7.26 CDCl3
4.89
4.88
4.72
4.71
4.19
4.18
4.17
4.15
1.46
1.45
1.44
0.95
1.00
1.05
1.04
0.95
0.96
2.04
2.94
f1 (ppm)
TfO
N
OEt
17

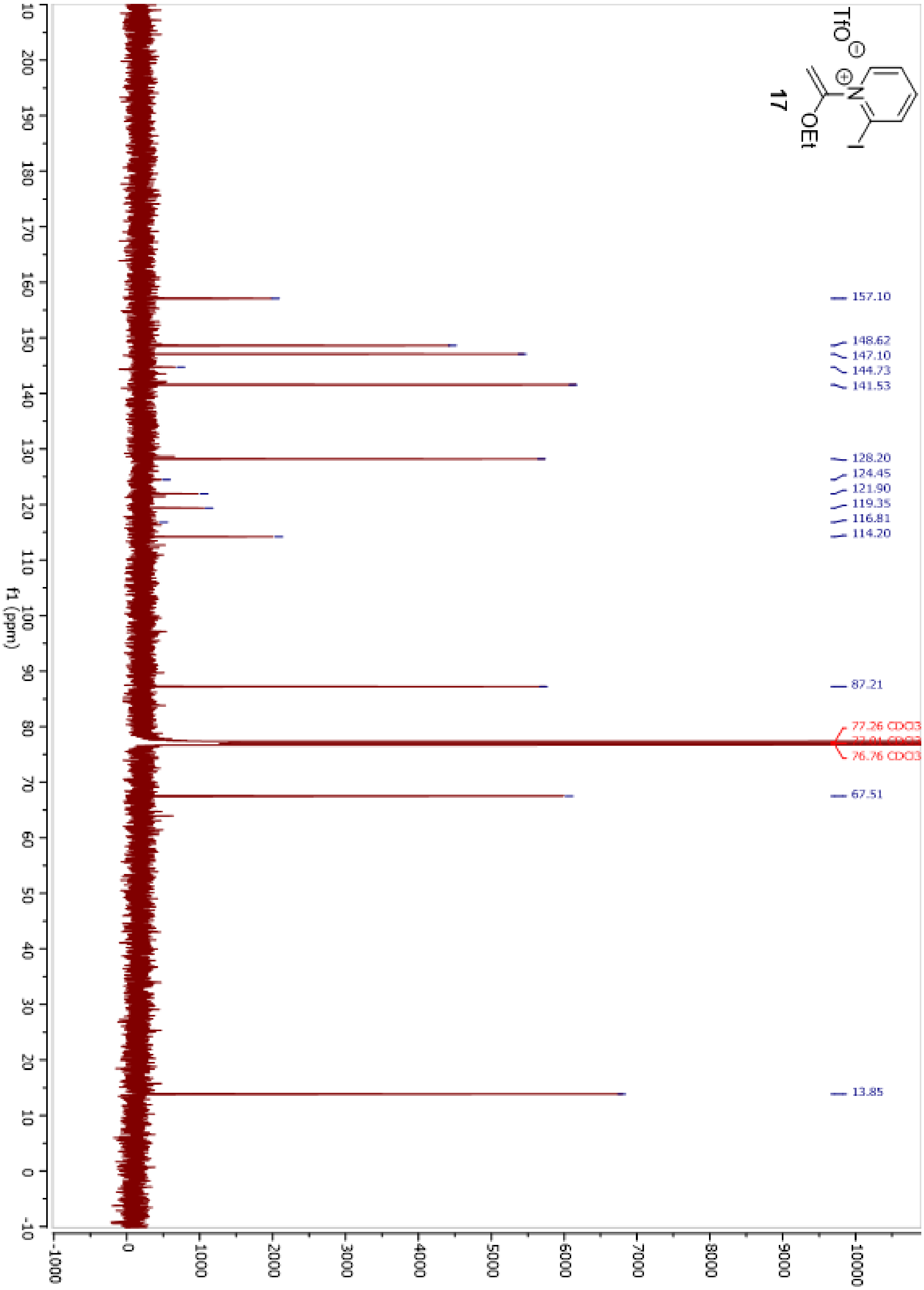
TfO
OEt
17
157.10
148.62
147.10
144.73
141.53
128.20
124.45
121.90
119.35
116.81
114.20
87.21
77.26 CDCl3
76.76 CDCl3
67.51
13.85
f1 (ppm)

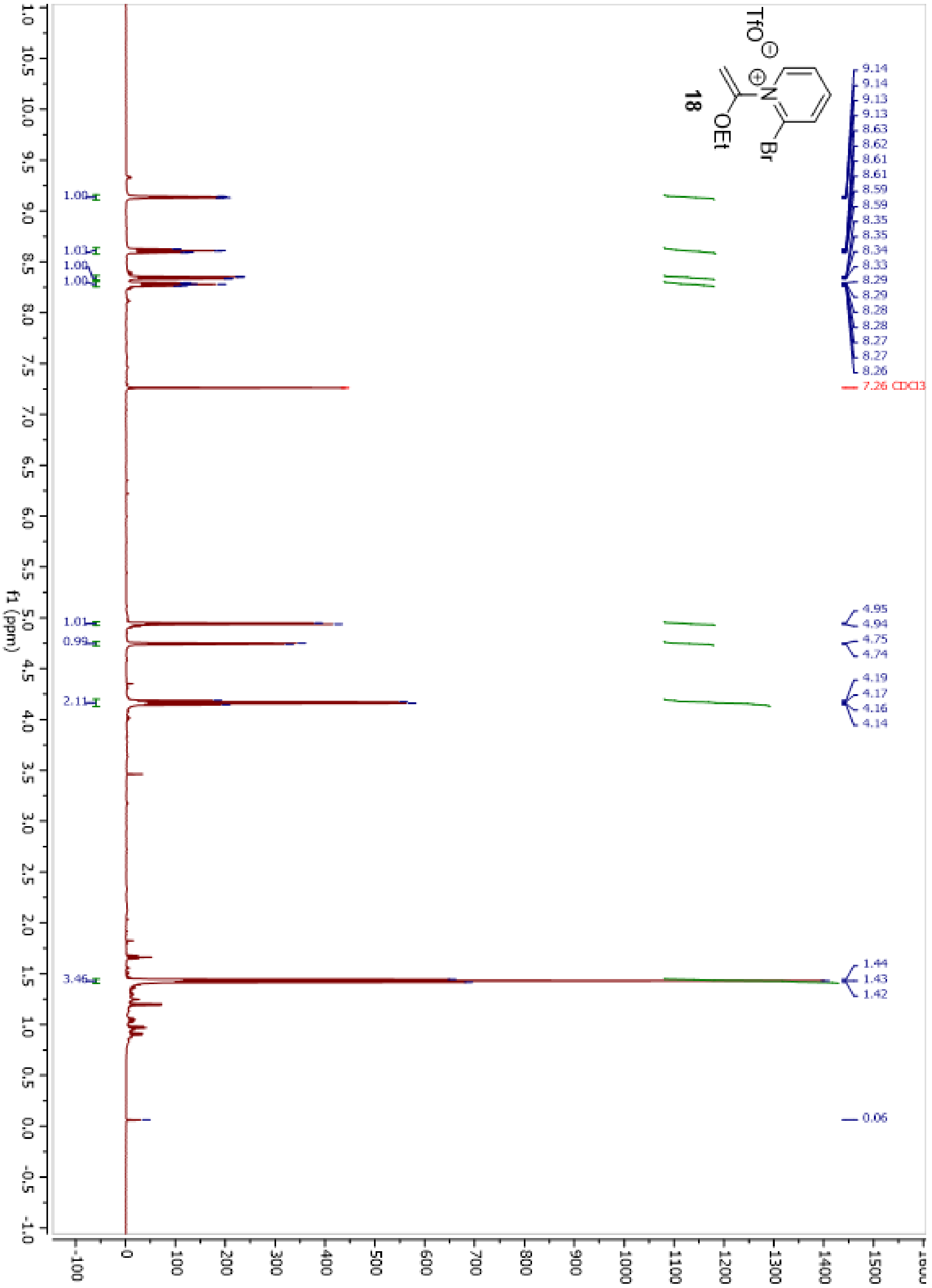

TfO
Br
OEt
18
f1 (ppm)
7.26 CDCl3

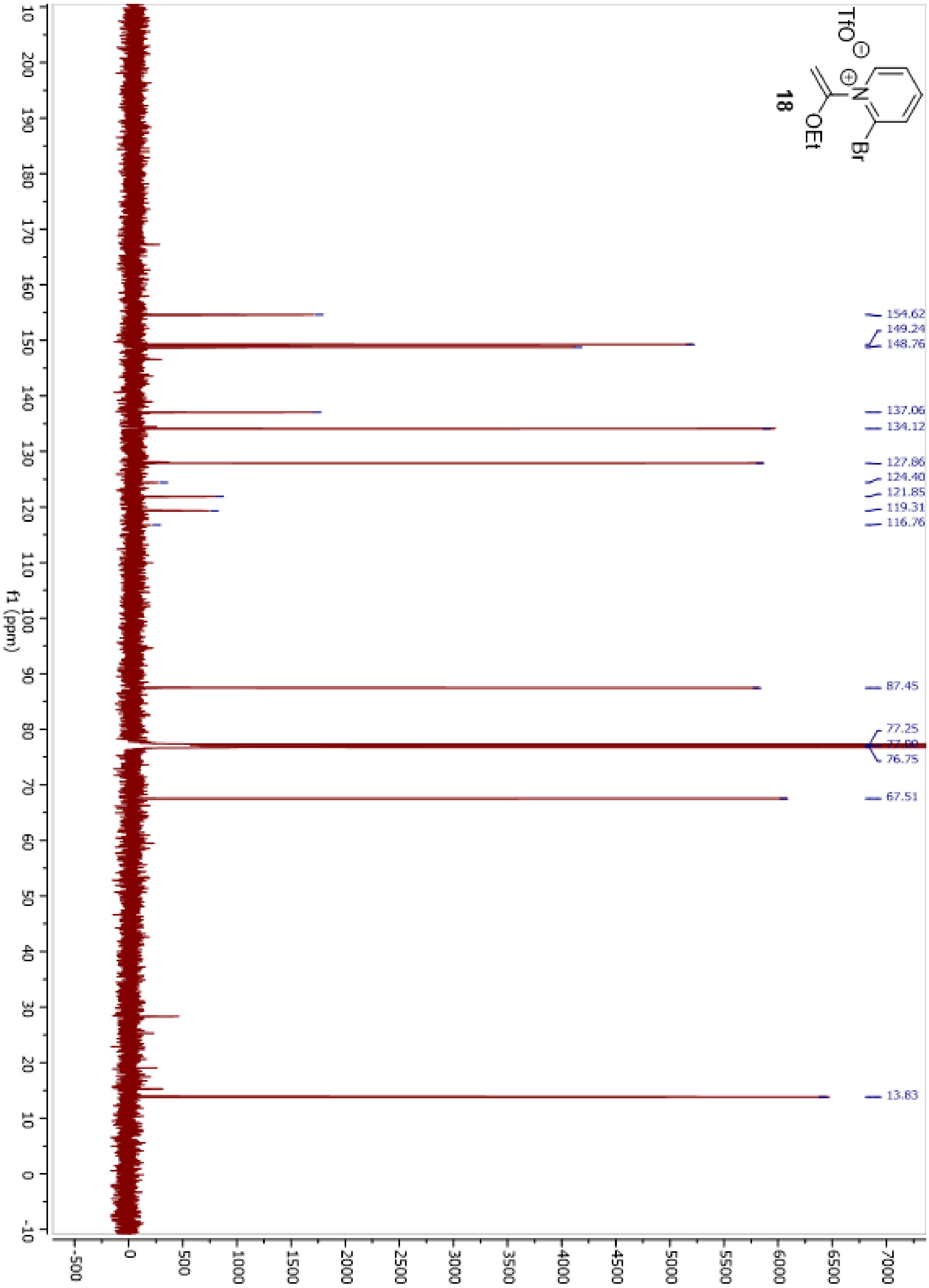
TfO
Br
OEt
18
154.62
149.24
148.76
137.06
134.12
127.86
124.40
121.85
119.31
116.76
87.45
77.25
76.75
67.51
13.83
f1 (ppm)

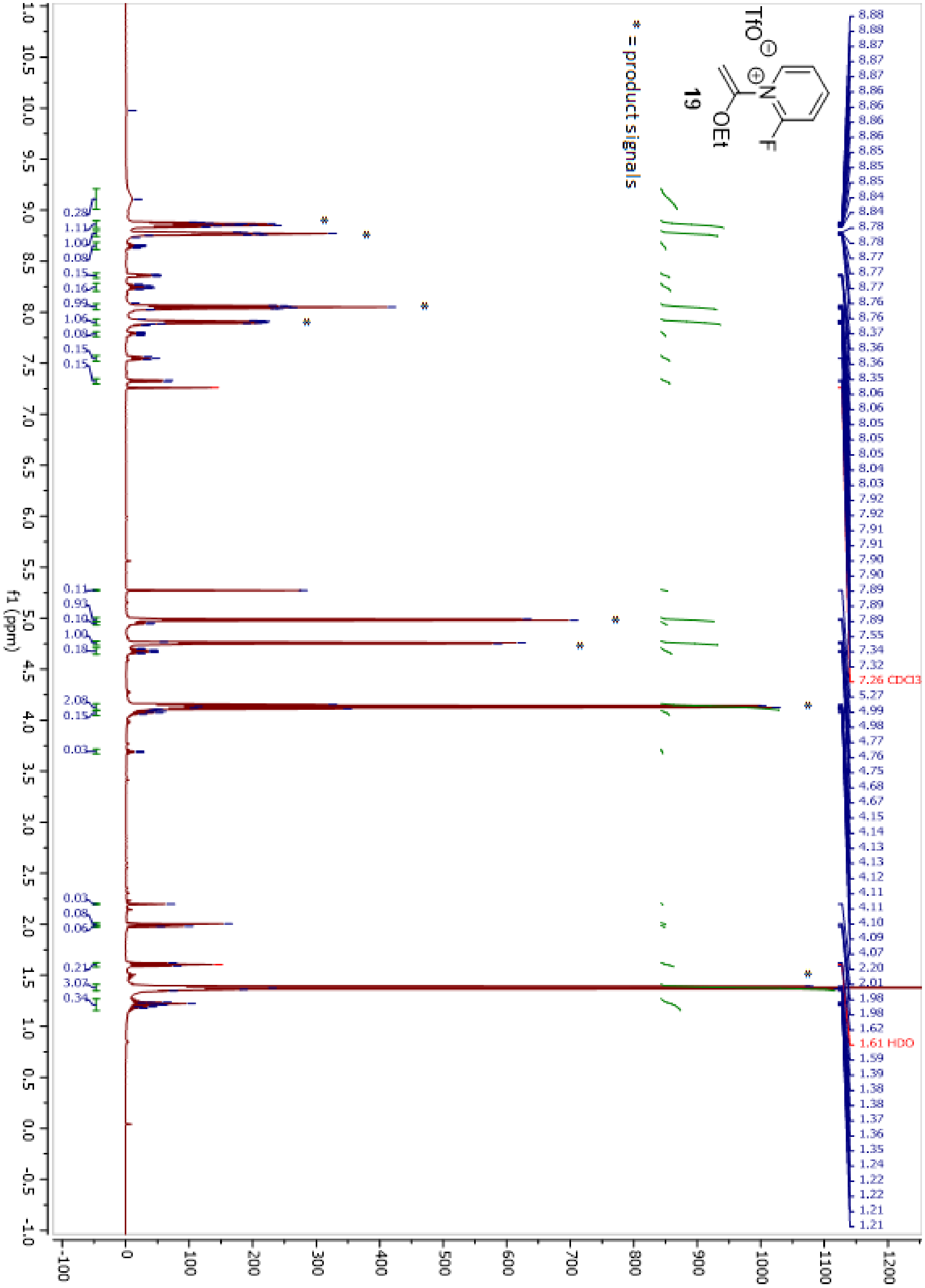

* = product signals
19
TfO
OEt
F
N
f1 (ppm)
7.26 CDCl3
1.61 HDO

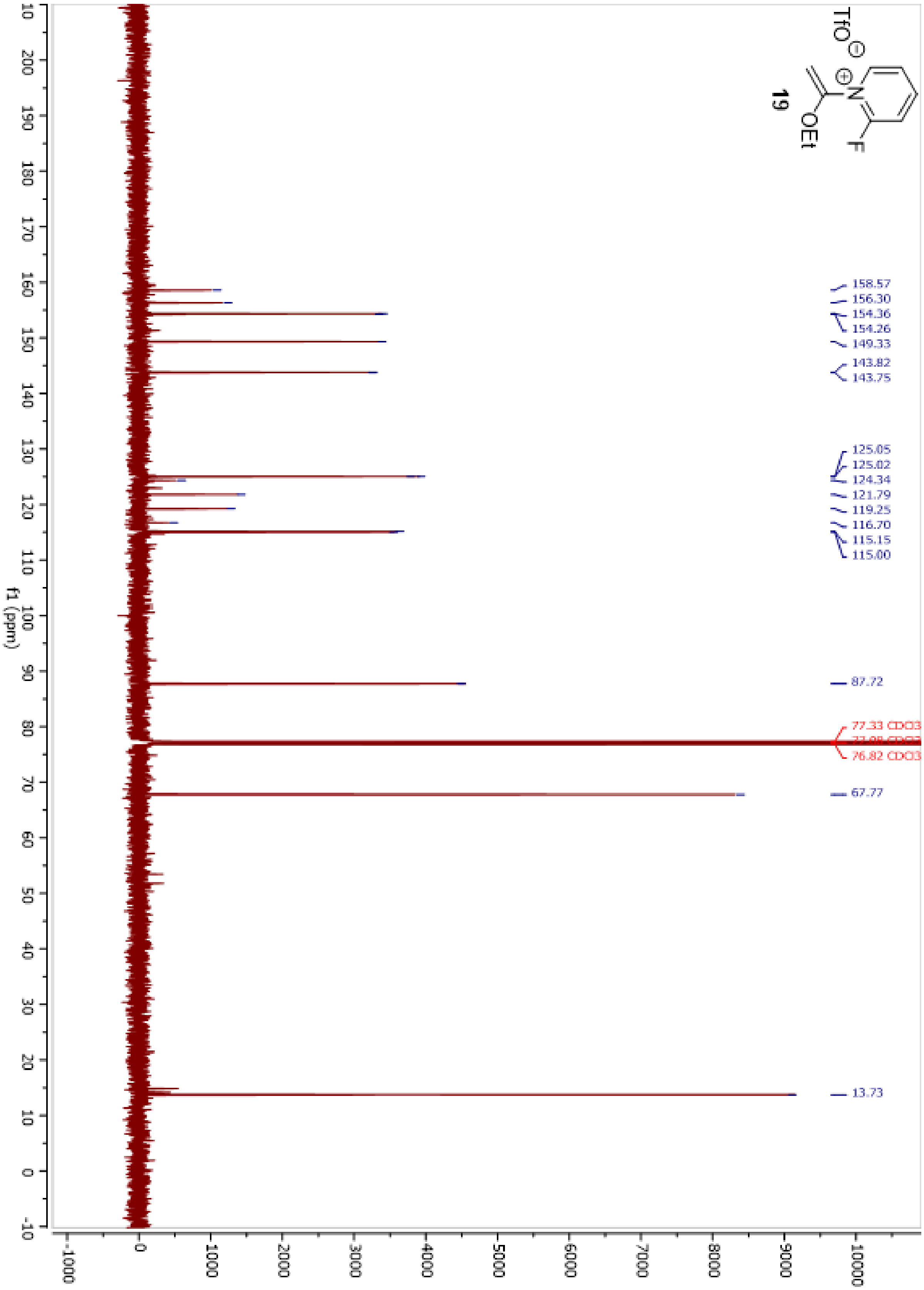
TfO
OEt
F
19
158.57
156.30
154.36
154.26
149.33
143.82
143.75
125.05
125.02
124.34
121.79
119.25
116.70
115.15
115.00
87.72
77.33 CDCl3
76.82 CDCl3
67.77
13.73
f1 (ppm)

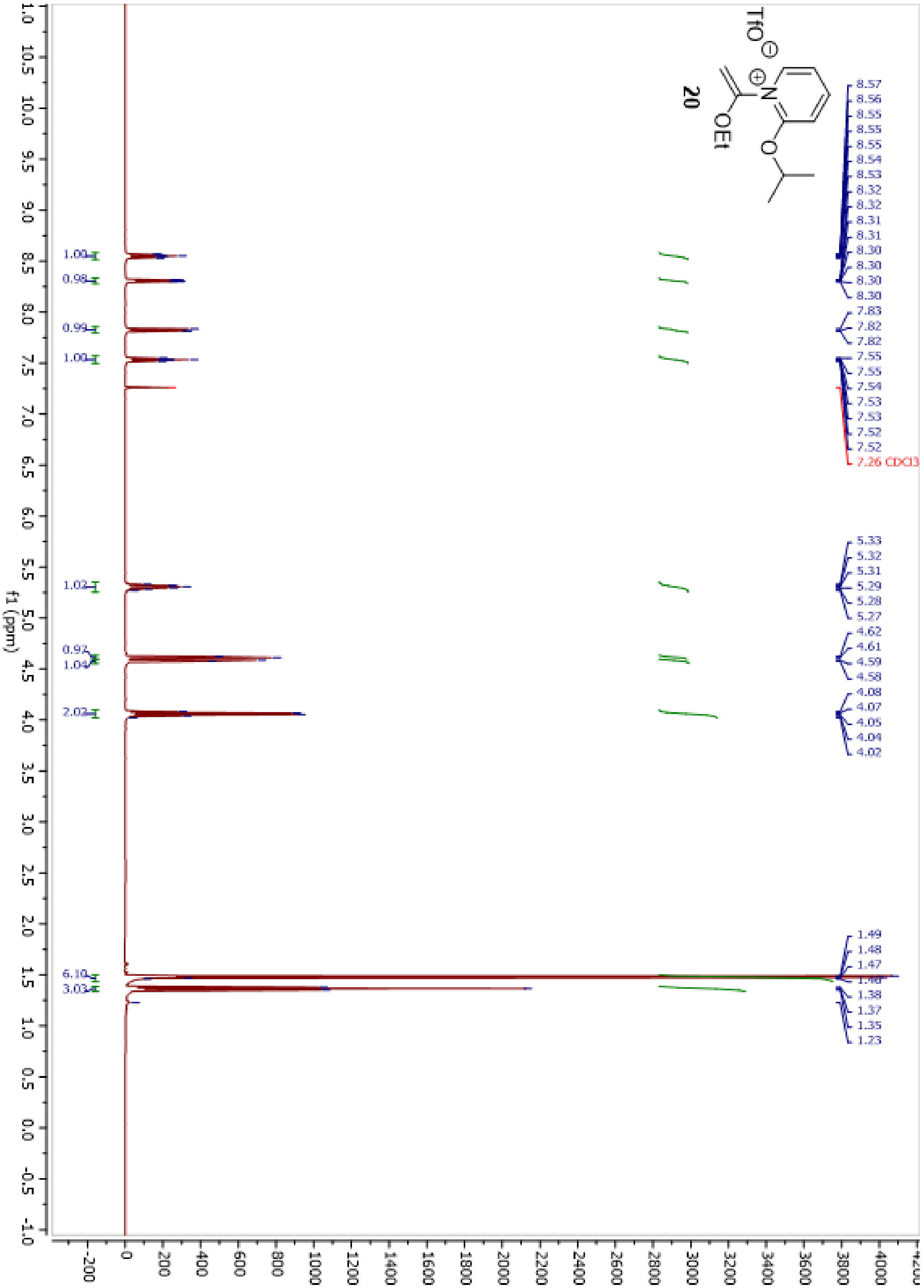

TfO
N
OEt
20
8.57
8.56
8.55
8.55
8.55
8.54
8.53
8.32
8.32
8.31
8.31
8.30
8.30
8.30
8.30
7.83
7.82
7.82
7.55
7.55
7.54
7.53
7.53
7.52
7.52
7.26 CDCl3
5.33
5.32
5.31
5.29
5.28
5.27
4.62
4.61
4.59
4.58
4.08
4.07
4.05
4.04
4.02
1.49
1.48
1.47
1.38
1.37
1.35
1.23
1.00
0.98
0.99
1.00
1.02
0.97
1.04
2.02
6.10
3.03
f1 (ppm)

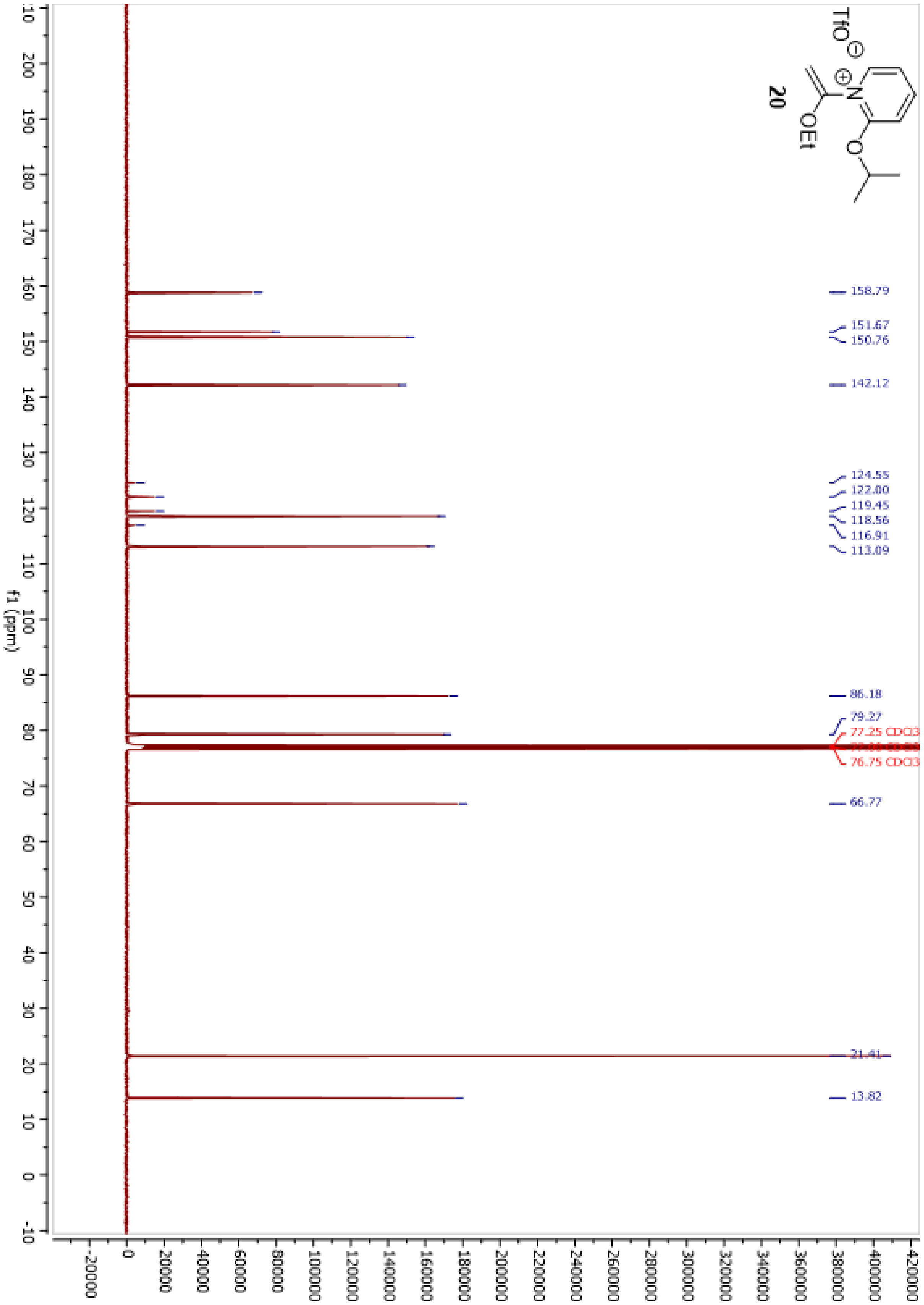

TfO
OEt
20
158.79
151.67
150.76
142.12
124.55
122.00
119.45
118.56
116.91
113.09
86.18
79.27
77.25 CDCl3
76.75 CDCl3
66.77
21.41
13.82
f1 (ppm)

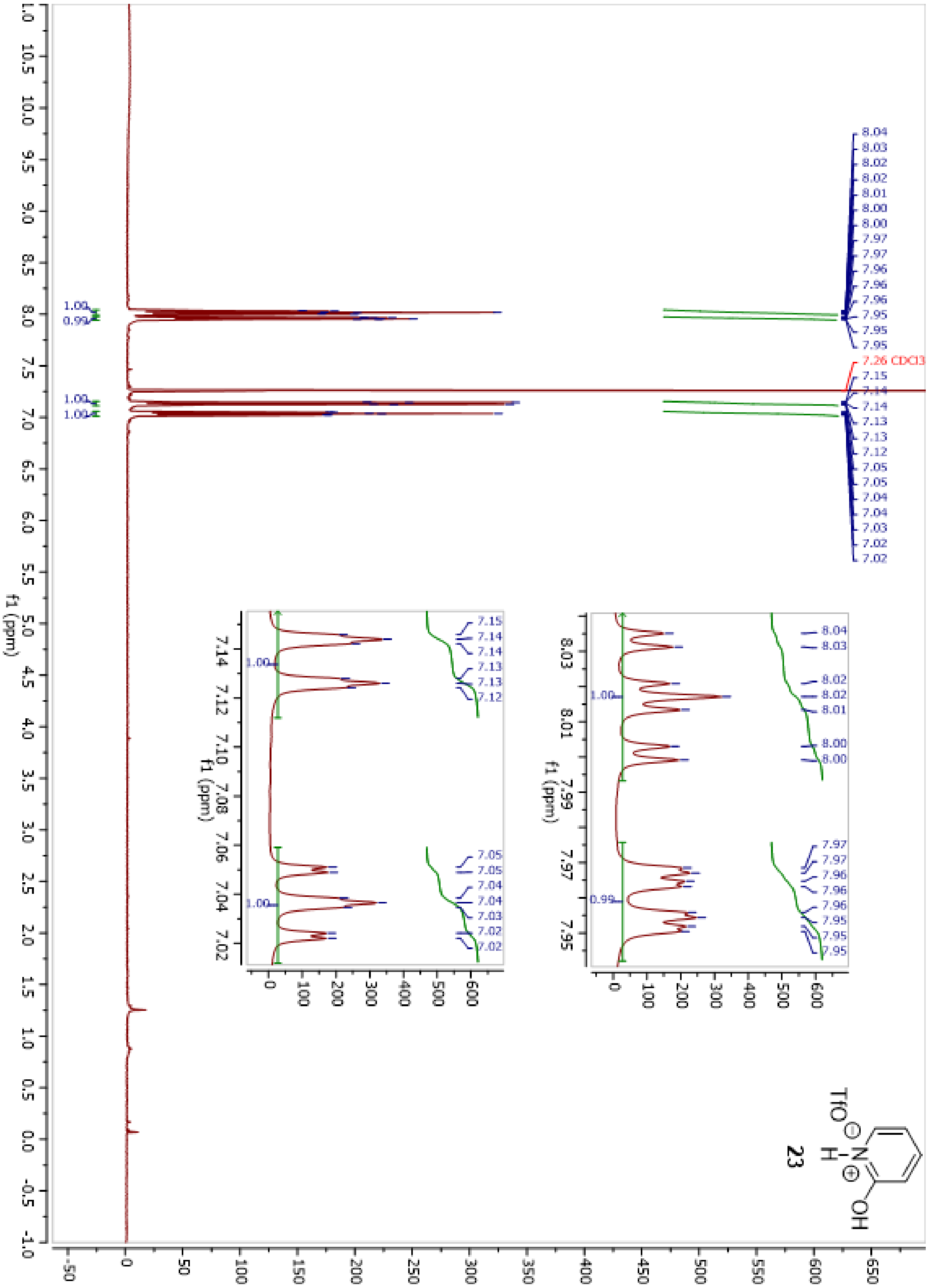
f1 (ppm)
7.26 CDCl3
TfO
OH
23

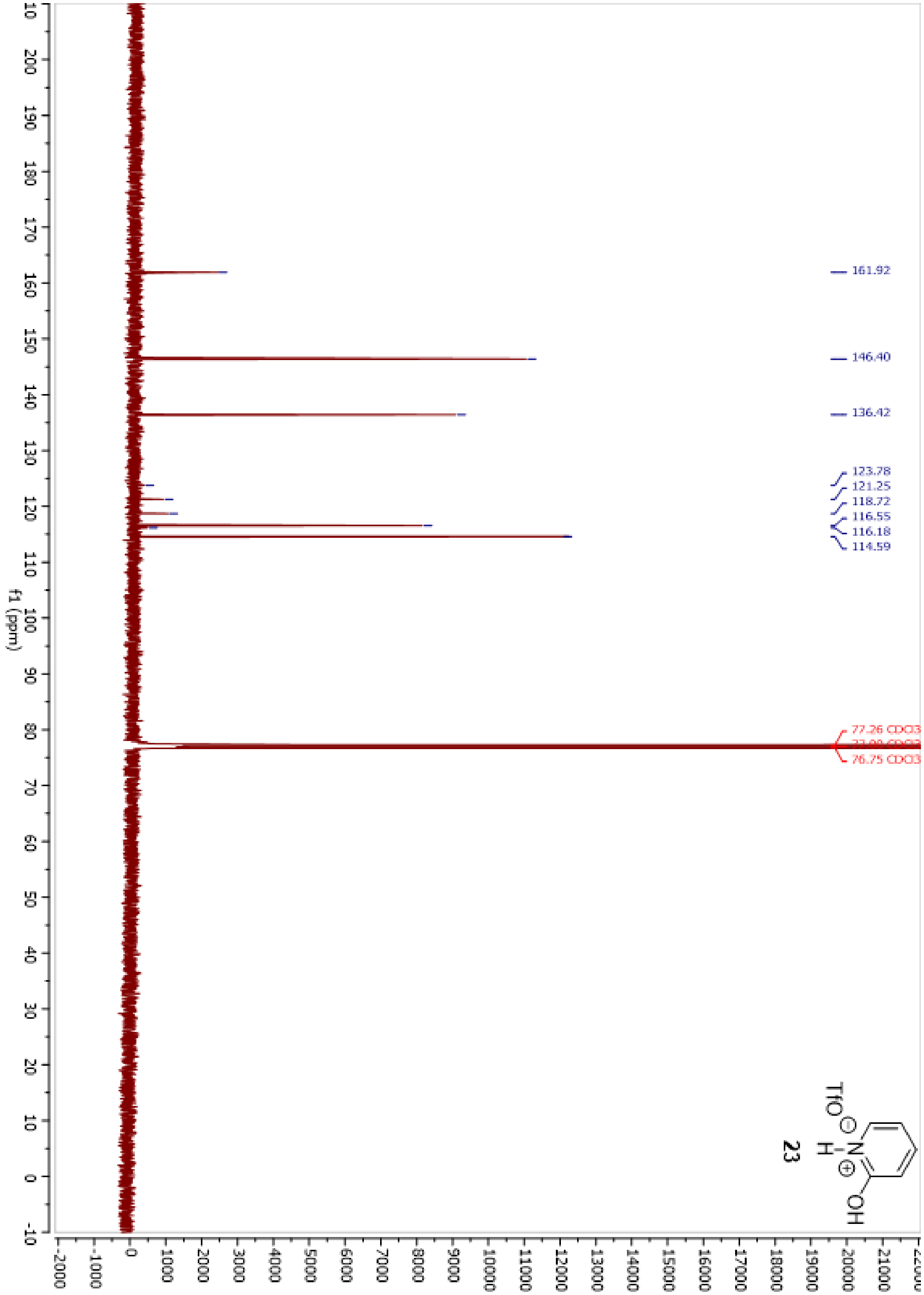
161.92
146.40
136.42
123.78
121.25
118.72
116.55
116.18
114.59
77.26 CDCl3
76.75 CDCl3
f1 (ppm)
TfO
OH
23
